# "Digital global health diplomacy" for climate change and human security in the Anthropocene

**DOI:** 10.34172/hpp.2022.35

**Published:** 2022-12-10

**Authors:** Vijay Kumar Chattu

**Affiliations:** ^1^ReSTORE Lab, Department of Occupational Science & Occupational Therapy, Temerty Faculty of Medicine, University of Toronto, Toronto, ON, Canada; ^2^Center for Transdisciplinary Research, Saveetha Dental College, Saveetha Institute of Medical and Technical Sciences, Saveetha University, Chennai, 600077, India; ^3^Department of Community Medicine, Faculty of Medicine, Datta Meghe Institute of Medical Sciences, Wardha, 442107, India

**Keywords:** Global Health, International health regulations, Diplomacy, Security, COVID-19, Climate change, Digital technology, Health policy

## Abstract

The COVID-19 pandemic has now affected everyone, threatening every aspect of our well-being with over 617597680 confirmed cases, including 6532705 deaths globally. The context of the Anthropocene is the backdrop for the novel, interlinked, systemic, and global threats. Anthropocene is a term proposed to designate the era in which human beings have become predominant drivers of planetary change, drastically altering the planet’s biosphere. The concept of global health diplomacy (GHD), which connects the domains of health and international relations, has a critical role in advancing human security. Thus, there is a need for new forms of diplomacy, which is critically important in this complex intermestic and interdependent Anthropocene era, where globalization has inevitably linked nations and population health. This paper introduces, analyzes, and attempts to define "Digital Global Health Diplomacy" (DGHD), which has gained great momentum during this COVID-19 pandemic with concurrent health and human security threats. The application of digital formats to the existing traditional structures for dialogue has become a more popular tool recently. Furthermore, digital means are being used during the COVID-19 pandemic to share the health diplomacy discourse at subnational, supranational, international, regional, and global platforms. DGHD reminds us again of the criticality of this multidisciplinary concept involving the contributions of diplomats, global health specialists, digital technology experts, economists, trade specialists, international law, political scientists, etc., in the global policymaking process. If used effectively by trained global health diplomats through innovative digital platforms, DGHD has a great scope of delivering results faster and has more reach than the traditional approach.

## Introduction

 The COVID-19 pandemic has now affected everyone, threatening every aspect of our well-being and instilling widespread fear globally. According to the World Health Organization (WHO), as of 26 October 2022, there have been 625 740 449 confirmed cases, including 6 563 667 deaths due to COVID-19 globally.^1^ The United Nations Development Programme’s (UNDP’s) 2022 Human Development report highlights that globally, more than 6 in 7 (85%) people perceived feeling either moderate or very insecure before the COVID-19 pandemic.^2^ The UNDP’s 1994 Human Development Report refocused the concept of human security from territorial to people’s security. It was endorsed by United Nations General Assembly in 2012, emphasizing everyone’s right to “freedom from fear,” “freedom from want,” and “freedom from indignity”.^3^ The context of the Anthropocene is the backdrop for the novel, interlinked, systemic, and global threats. These threats are related to digital technologies, violent conflict, inequalities among various social groups, and inadequacies in our healthcare systems. Therefore, as citizens in this globalized era, our efforts to find effective solutions for development problems ultimately result in those actions that mount planetary pressures. For example, despite all the development and technological advances, we continue to rely on fossil fuels resulting in increased greenhouse gas emissions worsening climate change, causing storms, floods, heatwaves, loss of biodiversity, and other zoonotic diseases, of which COVID-19 is the most recent one.^4^

 “Human security is an intrinsic complement to human development in the Anthropocene context. Permanent and universal attention to the next generation of human security can end the development of human insecurity pathways that produce pandemics, climate change, and the broader predicaments of the Anthropocene”.^2^ Thus, we need new forms of diplomacy (including digital), which is critically important in this complex intermestic and interdependent Anthropocene era, where globalization has inevitably linked nations and population health. In this context, global health diplomacy (GHD) plays a very significant role in addressing human security issues of the Anthropocene. For instance, the WHO has highlighted that health diplomacy can achieve the following goals, namely- (1) ensuring better health security and population health; (2) improving the relations between states; (3) having a commitment to improving health through the involvement of a wide range of actors, and (4) achieving outcomes that support poverty reduction and decreasing inequities.^5^ Besides, to substantiate the interconnection, a few authors have argued that although health diplomacy and human security are separate entities, there is some overlap as they both aim to protect human lives and safeguard human rights and human dignity.^6^ Therefore, GHD, which connects the domains of health and international relations, has a critical role in advancing human security.^7^ Given this context, this paper introduces, analyzes, and attempts to define the term “Digital Global Health Diplomacy” (DGHD), which has gained great momentum during this COVID-19 pandemic with concurrent health and human security threats.

## Human security challenges in the Anthropocene era

**Figure 1:**
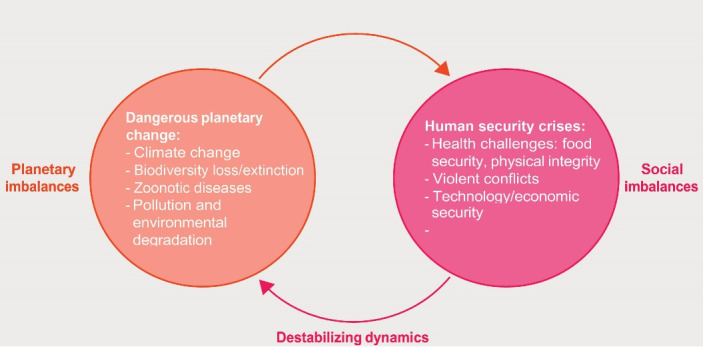
Representation of human security threats of the Anthropocene era. Source:Human Development Report Office^2^

 The concept of human security emerged, placing the security of human lives at the forefront of national and international security policies; a multi-sectoral, context-specific, and prevention-focused interdisciplinary normative framework that strongly emphasizes the protection and empowerment of the people.^7^ Anthropocene is a term proposed to designate the era in which human beings have become predominant drivers of planetary change, drastically altering the planet’s biosphere. Thus, there is a good reason to become insecure because numerous threats and challenges from the COVID-19 pandemic, climate change, biodiversity loss, and digital technology have taken new forms in recent years (Figure 1). The Anthropocene looms in the background of the divide between human progress and human security.^2^

**Figure 2:**
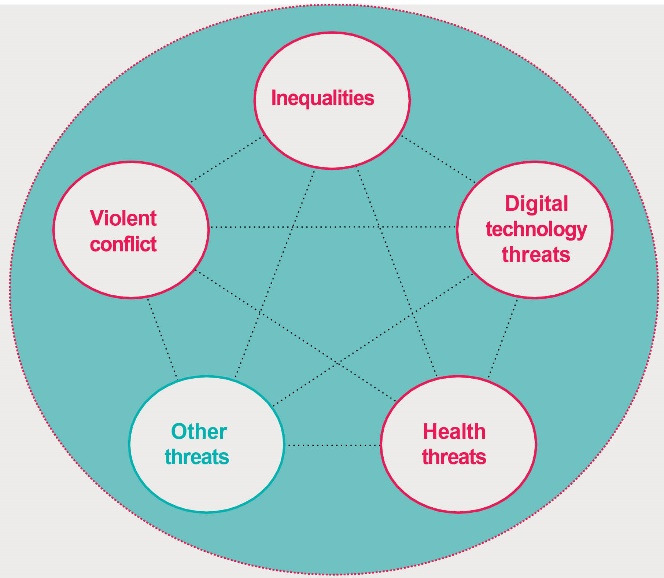
The new generation of human security threats. Source: Human Development Report Office^2^

 As the 2022 UNDP report highlights, the human security dimensions are multiple. As mentioned before, there are four main threats to human security that superimpose in the Anthropocene (Figure 2), namely: (*i*) the threats of digital technology, (*ii*) growing violent conflicts, (*iii*) expanding horizontal inequalities, and (*iv*) the growing challenges for healthcare systems. These complex threats are novel in their expression that they develop in the Anthropocene context, and their interconnectedness has been growing over time.^2^

- *Digital technologies-* Though the digital revolution has made human life easy, the rapid growth of digital development comes with novel threats that may exacerbate ongoing problems. Currently, the ongoing COVID-19 pandemic accelerated a digital shift in the growing economy everywhere, but simultaneously, cybercrime events have also skyrocketed, with annual losses projected to be around $6 trillion by December 2021.
- *Violent conflict*- Violent conflict events have been on the rise globally. As per the latest reports, around 1.2 billion (15% of the world’s population) live in conflict-affected areas, with around 560 million living outside fragile settings, reflecting the spread of various forms of violent conflict.^2^
- *Inequalities-*The inequalities in any form and anywhereare an assault on human dignity. LGBTQI (lesbian, gay, bisexual, transgender, queer and intersex) and other sexual minorities often face threats and particular risks of harm. Among 193 countries, 87% do not provide the LGBTQI identity with the right of recognition and full citizenship. For example, in 2020, around 47 000 women and girls were intentionally killed by their intimate partners or family members. On average, every 11 minutes, a woman or girl is slain by an intimate partner or a relative.
- *Climate change-*Climate change is a growing global crisis having stronger and long-lasting impacts, directly and indirectly, affecting people’s mental health and psychosocial well-being. Besides, it exacerbates multiple social and environmental risk factors of mental health, leading to anxiety and emotional distress.^8^ Globally, around 40 million people could die due to Climate change, mostly in developing countries, due to higher temperatures by the end of the century.^2^ The WHO estimates an excess of 250 million deaths between 2030 and 2050 due to the impacts of climate change.^9^

## GHD and the success stories

 GHD as a discipline bridges health and international relations domains by playing a key role in strengthening human security.^7^ A recent analysis of GHD by Taghizade et al^10^ has underscored its critical role in addressing a wide range of global challenges by formulation of certain key policies such as the WHO’s International Health Regulations 2005, the global Framework Convention of Tobacco Control, the universal health coverage,^11^ the UN Sustainable Developmental Goals, the 2019 UN Climate Change Conference (COP25) and the COVAX Facility (vaccine pillar of Access to COVID-19 Tools Accelerator) in 2020 to address the COVID‐19 pandemic.^12^

 Besides, the evolution of the Global Health Security Agenda committed to a safer world, the high-level Political Declaration by the United Nations on the Prevention and Control of Non-communicable diseases (NCDs), and the WHO’s Global Action Plan for the Prevention and Control of NCDs in the recent years^13^ are few more success stories of GHD. Various authors and researchers have emphasized GHD’s role in promoting peace,^14^ improving health, peace, and well‐being.^15^ In light of the COVID-19 pandemic, many authors in this field have emphasized GHD’s role in strengthening global leadership and international cooperation,^16^ global coordination,^17^ addressing commercial determinants of health and trade,^18^ negotiating for TRIPS waiver for COVID‐19 vaccines,^19^ promoting vaccine equity^20^ and strengthening the bonds between nations through vaccine diplomacy.^21^

## Evolution and the expanding scope of DGHD

 This paper introduces, for the first time, the term “Digital Global Health Diplomacy,” which incorporates all the actions and activities performed by the states/diplomats using digital technology applications (Zoom, Webex, Google, Microsoft teams, etc.) and social media platforms ( Twitter, Facebook, etc) to promote health & security or achieve better health outcomes or address the goal of securing global health agenda. Thus, applying the digital formats to the existing traditional structures for dialogue and sharing the health diplomacy discourse at subnational, supranational, international, regional, and global platforms has become a more practical and popular tool. E.g., most of the world leaders and government officials of 163 countries and 132 foreign affairs officials (Ambassadors) have accounts on Twitter.^22^

 To strengthen the above argument, during this COVID pandemic, we all have witnessed that communication channels have changed increasingly toward digital platforms, and Twitter’s role as a conversation forum has evolved. It is currently a vital tool for digital diplomacy, particularly when dialogue, peace-building efforts, and the development of regional cooperation are required. It serves as a vital link between diplomats and their different audiences, as well as between governments. Twitter came to the rescue of diplomats and world leaders as it became a platform for instant, interactive, and direct communication. Thus, Twitter emerged as the main medium for instant feedback, providing constructive criticism, and amplifying government messages.^22^ As Kickbusch highlights, the alias “Twitter Health diplomacy” also appears appropriate, as a quick search for “#healthdiplomacy” on social media platforms.^12^

 Recently, Godinho et al have proposed “Digital Health Diplomacy,” which highlights the use of “digital means for achieving health diplomacy goals”,^23^ which plays a significant role at sub-national, national, and regional levels. However, the definition and concept of “DGHD” align with the widely accepted definition of GHD, which refers to a multi‐level and multi‐actor negotiation process that shapes and manages the global policy environment for health in health and non-health forums.^24^ However, in this context of DGHD, the negotiations and diplomatic activities are conducted remotely/virtually through digital technologies using various software applications, social media, and dedicated #hashtags for agenda setting, promoting health, and strengthening human security.

 Digital diplomacy entails using digital technology by states, notably social media platforms such as Twitter and Facebook. A study by Garud-Patkar on the digital diplomacy of India towards South Asia concluded that some prominent Indian policy agendas on various social media platforms correlated with the agendas of the ‘foreign’ followers of South Asia— which indicates an agenda-building. Similarly, the prominent agendas from the region posted on social media were also aligned with India’s foreign policy priorities for the South Asian region, indicating effective digital diplomacy in progress.^25^ Because of the COVID-19 pandemic, currently, digital diplomacy notably supports states in conveying their foreign policies to domestic and international audiences. Another study on this domain by Sharma^22^ reported that digital diplomacy is critical to advancing a diplomatic agenda, where most (98%) of the 193 UN member-states regularly use Twitter and consequently have a wider social media presence. Besides, since 2020, we have observed many global summits, regional meets, G7 and G20 summits conducted online using the internet and various software due to travel bans, border closures, and travel restrictions that restricted in-person visits.

 Recently, Sharfi, a diplomat in the Gulf Cooperation Council, highlighted that the financial push for artificial intelligence (AI) in healthcare would positively impact GHD.^26^ However, it is also anticipated that emerging technologies, such as AI, if applied in GHD, may pose greater challenges in the future.^27^ Therefore, it is to be noted that digitally assisted GHD can assist agenda-setting, sharing common values and interests, and aligning with regional/global policies. As highlighted by Taghizade et al, GHD played a key role in strengthening international cooperation, strengthening health systems, improving the global economy & trade, and addressing the inequities for achieving health-related global targets by using digital platforms during this COVID-19 pandemic.^10^ Thus, even by practicing DGHD in this multipolar world, there is a great scope to address several complex issues in this Anthropocene era with interlinked and complex geo-socioeconomic and political determinants.

## Conclusions

 In this Anthropocene era, amid various human security threats around, the art of diplomacy is forced to consider technology as an indispensable partner for achieving sustainable development goals. The concept of DGHD is a new mode of communication that is in its infancy and is playing a very critical role in addressing a wider spectrum of global issues. These issues range from the pandemic, infodemic management, saving the world from misinformation about vaccines, climate change, addressing equity gaps, strengthening bonds between nations, and policy development for various health crises. The concept of DGHD reminds us again of the criticality of its multidisciplinary nature through the contributions of diplomats, global health specialists, digital technology experts, economists, trade specialists, international law, political scientists, etc., in the global policymaking process. DGHD, if used effectively by trained global health diplomats, has a great scope of delivering results faster and has more reach than the traditional approach by utilizing innovative digital platforms.

## Acknowledgments

 This paper was done as part of the School of Modern Diplomacy program jointly conducted by the United Nations University- Institute on Comparative Regional Integration Studies (UNU-CRIS)- Belgium, and Vienna School of International Studies-Austria, in September 2022. The author thanks all the faculty involved in Climate Diplomacy, Science & Health Diplomacy, and Digital Diplomacy for sharing useful resources.

## Funding

 The study did not receive any funding.

## Ethical Approval

 Not applicable.

## Competing Interests

 VKC is an editorial board member in *Health Promotion Perspectives*.
